# Molecular characterization of three digeneans parasitizing marine fish in the Gulf of Thailand

**DOI:** 10.3389/fvets.2025.1694904

**Published:** 2025-12-23

**Authors:** Nannaphat Suwannarat, Carmen Gilardoni, Alexis Ribas, Jordi Miquel, Srisupaph Poonlaphdecha

**Affiliations:** 1 Program in Fishery Science and Aquatic Resources, Department of Agricultural Technology, King Mongkut’s Institute of Technology Ladkrabang, Prince of Chumphon Campus, Chumphon, Thailand; 2 Parasitology Section, Department of Biology, Healthcare and Environment, Faculty of Pharmacy and Food Science, University of Barcelona, Barcelona, Spain; 3 Institute of Research in Biodiversity (IRBio), University of Barcelona (UB), Barcelona, Spain

**Keywords:** Lecithocladium, Prodistomum, Lepocreadioides, Rastrelliger, Paraplagusia, 18S, ITS1, 28S

## Abstract

The Gulf of Thailand is a highly diverse marine ecosystem, yet knowledge of its helminth fauna remains fragmentary. Marine fishes were obtained in 2024 from accidental catches of crab-trap fishers or captured by local fishermen using boats in Chumphon Province, Thailand. In this study, we molecularly characterize three digenean trematode genera parasitizing marine fishes from this area: *Lecithocladium* (Hemiuridae) from the short mackerel (*Rastrelliger brachysoma*), with a prevalence of 77% in 22 fishes and ten worms examined for morphology; *Prodistomum* (Lepocreadiidae) from *R.brachysoma*, with a prevalence of 18% in 22 fishes, and from *Rastrelliger kanagurta*, with a prevalence of 100% in one fish, with 10 worms examined for morphology; and *Lepocreadioides* (Lepocreadiidae) from the double-lined tonguesole (*Paraplagusia bilineata*), with a prevalence of 75% in four fishes, and eight worms examined for morphology. Sequences of nuclear ribosomal markers (18S, ITS2, and 28S) were generated, providing the first molecular data for *Lepocreadioides orientalis*. Our analyses confirm the presence of *Lecithocladium angustiovum* and *Prodistomum* sp. in the Gulf of Thailand, contribute new molecular data, and provide a morphological description of *Prodistomum* sp. Despite the Gulf of Thailand’s high ichthyofaunal diversity, its helminth communities remain insufficiently investigated, highlighting the need for future research.

## 1 Introduction

The Gulf of Thailand ecoregion, a semi-enclosed sea in Southeast Asia, is part of the Central Indo-Pacific Realm and the Sunda Shelf Province (1). This ecoregion is characterized as a relatively shallow marine basin, with a mean depth of approximately 50 m and an estimated surface area of 350,000 km^2^ within the Southeast Asian maritime region. Paleoceanographic reconstructions indicate that, during the late Pleistocene, approximately 12,000 years before the present, global sea levels were 100–150 m lower than current levels. Consequently, the present-day Gulf of Thailand constituted part of the emergent Sundaland subcontinent. This implies that the fish species presently inhabiting the Gulf of Thailand colonized the area relatively recently, having dispersed from regions beyond Sundaland. A total of 372 commercial fish species belonging to 109 families have been identified in the Gulf of Thailand (2). However, this previous study is an underestimation of the region’s marine fish biodiversity, as non-commercial species were not included. A recent bottom otter-trawl survey (30–58 m) in the upper Gulf of Thailand found 131 species from 52 families, including 2 new records (3). The method captures both commercial and non-commercial fish, but is limited to the surveyed muddy habitats (3).

In general, studies on the helminths of marine fishes in the Gulf of Thailand are quite limited relative to its high marine fish diversity, and previous helminthological data on digenean trematodes (our literature search did not include ectoparasites from gills) are really limited. The hemiurid *Lecithocladium cristatum* (Rudolphi, 1819; Looss, 1907) was isolated from the carangid *Parastromateus niger* (Bloch, 1795) and was molecularly characterized following the procedure shown in (4) using 18S rRNA. A recent study on the short mackerel [*Rastrelliger brachysoma* (Bleeker, 1851)], a commercially important species highly preferred by Thai consumers, provided the first data on digenean trematodes in this host species from the Gulf of Thailand. Four digenean trematodes (*Lecithocladium* sp., *Prodistomum* sp., *Opechona* sp., and *Aphanurus* sp.) were reported, and 28S rRNA sequences were generated to determine their phylogenetic position (5). A subsequent study on the same fish species from Chon Buri Province identified three unnominated species belonging to three different genera (*Lecithocladium* sp., *Opechona* sp., and *Prodistomum* sp.) but did not provide molecular data (6). To our knowledge, there are no additional published studies (including those in Thai-language scientific journals) on digenean trematodes of marine fishes in the Gulf of Thailand.

The presence of cryptic species is common among digenean trematodes, and accurate taxonomic resolution often requires the application of molecular tools, as demonstrated in marine fish parasites (7, 8). The objective of the present study is therefore to provide novel helminthological data on digenean trematodes from the Gulf of Thailand, a region where, despite its high ichthyofaunal diversity, helminth communities remain insufficiently investigated. According to the available studies, which cover only a few host species, the existing data are incomplete because they lack detailed morphological descriptions, do not identify specimens at the species level, or omit molecular data.

## 2 Materials and methods

Fishes were obtained from accidental catches by crab-trap fishers or captured by local fishermen using boats between July and August 2024 in Pathio district, Chumphon Province, Thailand. Identification of marine fishes was primarily based on the methods in (2, 9), with taxonomic nomenclature following (9). The fish specimens were examined for the presence of helminths immediately after collection at the Laboratory of the Department of Agricultural Technology, King Mongkut’s Institute of Technology, Ladkrabang, Prince of Chumphon Campus, Thailand. Helminths were isolated under a stereomicroscope using a seawater saline solution. Recovered helminths were preserved in absolute ethanol for subsequent molecular analyses, while a subset was fixed in a hot seawater saline solution, transferred to 70% ethanol, and later stained with Semichon’s acetocarmine. Stained specimens were observed by optic microscopy and identified to the lowest taxonomic level according to (10–13). A total of 10 individuals of a parasite species that had not been previously described were measured with a microscope-mounted digital camera (Leica DMLB). Measurements are presented in μm as average (minimum-maximum). One specimen of each species was deposited in the Museum of Natural Sciences of Barcelona (collection numbers MZB 2025-7,272 to MZB 2025-7,274). Prevalences were calculated as the number of parasitized fish over the total number of fish examined and expressed as a percentage (14).

Molecular studies of 2–3 specimens of each species were performed using rDNA (18S, ITS2, and 28S) markers. DNA was extracted using the DNeasy Blood & Tissue Kit (Qiagen, Germany) according to the manufacturer’s instructions. The 18S, ITS2, and 28S regions of the rDNA were amplified by PCR. The PCRs were performed in a total volume of 25 μL containing 1X Green GoTaq® Reaction Buffer (1.5 mM MgCl_2_), 0.2 mM of each dNTP, 1 μM of each primer, and 5 u/μL of GoTaq® DNA polymerase (Promega). The 18S region was amplified using the primers 18S F (5-ATCCGAAGTAATGGTTAAGAGGG-3) and 18S R (5-ACCTACGGAAACCTTGTTACG-3); the ITS2 region using ITS2 F (5-GCTCGTGTGTCGATGAAGAG-3) and ITS2 R (5-AGGCTTCGGTGCTGGGCT-3); and the 28S regions using the primers 28S F (5-GTGAATACCCGCTGAACTTAAGC-3) and 28S R (5-TCTCCTTGGTCCGTGTTTCAA-3) (15). The cycling conditions included an initial denaturation at 94 °C for 5 min, followed by 40 cycles of 30 s at 94 °C, 30 s at 52 °C for 28S and 18S, 56 °C for ITS2 (annealing), and 2 min at 72 °C, with a final extension step of 10 min at 72 °C. Amplified PCR products were electrophoretically separated in a 1% (w/v) agarose gel stained with Green Gel. Negative controls for the PCR were always run to check for contamination. Relevant bands were sent for purification and sequencing, and all sequences have been deposited in GenBank (Tables 1, 2). Sequencing was performed by the Genomics Unit of the Scientific and Technological Centers (CCiTUB), Universitat de Barcelona.

**Table 1:** Collection of data and GenBank accession numbers for Hemiuridae species included in phylogenetic analyses.

Species	Host	28S	Reference
*Pleural digitatus*	*Scomberomorus commerson*	AY222201	(34)
*Tubulovesicula laticaudi*	*Hydrophis cyanocinctus*	OR209733	(35)
*Lecithochirium floridense*	*Syacium papillosum*	MK558793	(36)
*Lecithochirium floridense*	*Percophis brasiliensis*	OP918131	(37)
*Lecithochirium synodi*	*Auxis thazard*	OP458331	(38)
*Lecithochirium synodi*	*Pseudopercis numida*	OP918132	(37)
*Lecithochirium microstomum*	*Trichiurus lepturus*	KC985235	(39)
*Lecithochirium microstomum*	*Trichiurus lepturus*	OP918120	(37)
*Lecithochirium microstomum*	*Prionotus punctatus*	OP918127	(37)
*Lecithochirium cf. muraenae*	*Gymnothorax vicinus*	OP918128	(37)
*Lecithochirium cyanovitellosum*	*Laticauda semifasciata*	MH628314	(40)
*Hemiurus levinseni*	*Cylichna alba*	MN962992	(41)
*Myosaccium ecaude*	*Sardinella brasiliensis*	OP918123	(37)
*Parahemiurus merus*	*Harengula clupeola*	OP918125	(37)
*Elytrophalloides oatesi*	*Notothenia coriiceps*	ON123030	(42)
*Dinurus euthynni*	*Auxis thazard*	OP458333	(38)
*Ectenurus virgula*	*Prionotus punctatus*	OP918126	(37)
*Dinurus longisinus*	*Coryphaena hippurus*	AY222202	(34)
*Lecithocladium excisum*	*Scomber scombrus*	AY222203	(34)
***Lecithocladium angustiovum***	***Rastrelliger brachysoma***	**PX138732**	**Present study**
***Lecithocladium angustiovum***	***Rastrelliger brachysoma***	**PX138733**	**Present study**
*Lecithocladium* sp.	*Rastrelliger brachysoma*	PP768938	(5)
*Lecithocladium* sp.	*Rastrelliger brachysoma*	PP768937	(5)
*Lecithocladium* sp.	*Rastrelliger brachysoma*	PP768939	(5)
*Lecithocladium* sp.	*Rastrelliger brachysoma*	PP768936	(5)
*Aphanurus* sp.	*Rastrelliger brachysoma*	PP768940	(5)
*Aphanurus mugilus*	*Moolgarda engeli*	LT607809	(43)
*Aphanurus mugilus*	*Moolgarda engeli*	LT607808	(43)
*Aphanurus mugilus*	*Moolgarda engeli*	LT607807	(43)
*Dinosoma synaphobranchi*	*Antimora microlepis*	MH628304	(40)
*Brachyphallus crenatus*	*Salvelinus leucomaenis*	MH628299	(40)
*Genolinea anura*	*Pleurogrammus monopterygius*	MH628308	(40)
*Genolinea bowersi*	*Notothenia coriiceps*	ON123031	(42)
*Robinia aurata*	*Liza aurata*	DQ354367	(44)
*Bunocotyle progenetica*	*Hydrobia ulvae*	DQ354365	(44)
*Saturnius gibsonii*	*Mugil cephalus*	KJ010542	(45)
*Saturnius* sp.	*Mugil cephalus*	DQ354366	(44)
*Merlucciotrema praeclarum*	*Cataetyx laticeps*	AY222204	(34)
*Aponurus* sp.	*Mullus surmuletus*	DQ354368	(44)
*Aponurus* sp.	*Mullus barbatus*	HQ713441	(46)
Outgroups			
*Echinostoma paraensei*	Hamster	EU025867	Unpublished
*Echinostoma miyagawai*	*Anas platyrhynchos*	KY436408	(47)

In bold new sequences obtained in the present study.

**Table 2:** Collection of data and GenBank accession numbers for Lepocreadiidae species included in phylogenetic analyses.

Species	Host	ITS2	28S	Reference
*Cephalolepidapedon warehou*	*Cyanea annaskala*	MT773345		(29)
*Cephalolepidapedon warehou*	*Seriolella brama*	MT773347		(29)
*Cephalolepidapedon saba*	*Aldersladia magnificus*, *Scomber japonicus*, *Scomber australasicus*	PP239547	PP239560-61	(13)
*Clavogalea trachinoti*	*Trachinotus coppingeri*	MH157057	MH157067	(31)
*Lepocreadium album*	Unknown	MK418259		Unpublished
*Lepocreadium oyabitcha*	*Abudefduf sordidus*	OM777008	OM777006	(23)
*Lepocreadium trulla*	*Lutjanus campechanus*, *Rhomboplites aurorubens*	KU527433	KU527432	(48)
*Opechona chloroscombri*	*Chloroscombrus chrysurus*		MZ345679	(49)
*Opechona austrobacillaris*	*Pomatomus saltatrix*	MH157063	MH157073	(31)
*Opechona corkumi*	*Peprilus burti*		MZ345683	(49)
*Opechona kahawai*	*Arripis trutta*	PP239538	PP239556	(13)
*Opechona olssoni*	*Scomber japonicus*		MT303947	(50)
*Opechona cf. olssoni*	*Aldersladia magnificus*, *Scomber australasicus*	PP239539	PP239555	(13)
*Opechona pharyngodactyla*	*Trachinotus rhodopus*		OQ676201	(51)
*Opechona* sp.	*Buccinanops cochlidium*	KF451939	KF451939	(52)
*Opechona* sp.	*Rastrelliger brachysoma*		PP768943	(5)
*Opechonoides opisthoporus*	*Pomacentrus moluccensis*, *Abudefduf whitleyi*	OM777017	OM777005	(23)
*Pelopscreadium spongiosum*	*Ostracion cubicus*		FJ788469	(53)
*Prodistomum keyam*	*Monodactylus argenteus*	MH157064	MH157074	(31)
*Prodistomum keyam*	*Pukia falcata*	PP239536		(13)
*Prodistomum orientale*	*Scomber australasicus*	MT773350	MT299625-26	(29)
*Prodistomum orientale* type 1	*Pukia falcata*	PP239534	PP239554	(13)
*Prodistomum orientale* type 1	*Scomber australasicus*	PP239525		(13)
*Prodistomum orientale* type 2	*Scomber japonicus*	PP239535	PP239553	(13)
*Prodistomum* sp. type 3	*Scomber australasicus*	MT773352	PP239552	(29)
*Prodistomum* sp. type 3	*Aldersladia magnificus*	PP239522		(13)
*Prodistomum* sp. type 3	*Chrysaora pentastoma*	PP239516		(13)
*Prodistomum* sp. type 5	*Scomber japonicus*	PP239546	PP239557	(13)
*Prodistomum* sp. type 4	*Scomber japonicus*	PP239545	PP239558-59	(13)
***Prodistomum* sp. SPK26**	***Rastrelliger kanagurta***	**PX137635**	**PX138247**	**Present study**
***Prodistomum* sp. SPK27**	***Rastrelliger brachysoma***	**PX137636**	**PX138248**	**Present study**
*Prodistomum* sp.	*Rastrelliger brachysoma*		PP768941-42	(5)
*Prodistomum priedei*	*Epigonus telescopus*		AJ405272	(54)
*Prodistomum alaskense*	*Zaprora silenus*		MT303950	(52)
Lepocreadiidae gen. sp. 1	*Crepipatella dilatata*	KF451933		(55)
Lepocreadiidae gen. sp. 2	*Pareuthria plumbea*	KF451935		(55)
Lepocreadiidae gen. sp. 3	*Scomber japonicus*	KF451937	KF451937	Unpublished
Lepocreadiidae sp.	*Rhizostoma pulmo*		OM910739	(56)
*Preptetos laguncula*	*Naso unicornis*	MZ702046	MZ701986	(8)
*Preptetos cannoni*	*Siganus punctatus*	MZ702037	MZ701993	(8)
*Preptetos prudhoei*	*Zebrasoma velifer*	MZ702071	MZ701995	(8)
*Preptetos zebravaranus*	*Zebrasoma scopas*	MZ702075	MZ701999	(8)
*Preptetos paracaballeroi*	*Naso vlamingii*	MZ702053	MZ702004	(8)
*Preptetos allocaballeroi*	*Naso annulatus*	MZ702030	MZ702002	(8)
*Preptetos quandamooka*	*Prionurus microlepidotus*	MZ702073	MZ702009	(8)
*Preptetos pearsoni*	*Prionurus maculatus*	MZ702058	MZ702007	(8)
*Lepotrema adlardi*	*Abudefduf bengalensis*	MH730000	MH730015	(57)
*Lepotrema amblyglyphidodonis*	*Amblyglyphidodon curacao*	MH730003	MH730017	(57)
*Lepotrema melichthydis*	*Melichthys vidua*	MH730008	MH730021	(57)
*Lepotrema moretonense*	*Prionurus microlepidotus*	MH730013	MH730023	(57)
*Lepotrema acanthochromidis*	*Acanthochromis polyacanthus*	MH729999	MH730014	(57)
*Lepotrema hemitaurichthydis*	*Hemitaurichthys polylepis*	MH730006	MH730020	(57)
*Lepotrema monile*	*Pomacentrus wardi*	MH730009	MH730024	(57)
*Lepotrema amansis*	*Amanses scopas*	MH730001	MH730016	(57)
*Lepotrema cirripectis*	*Cirripectes chelomatus*	MH730004	MH730018	(57)
*Mobahincia teirae*	*Platax teira*	MH157058	MH157068	(13)
*Lobatocreadium exiguum*	*Pseudobalistes fuscus*		FJ788484	(58)
***Lepocreadioides orientalis***	***Paraplagusia bilineata***	**PX138688**		**Present study**
*Multitestis magnacetabulum*	*Platax teira*	MH157061	MH157071	(31)
*Neomultitestis aspidogastriformis*	*Platax teira*	MH157062	MH157072	(31)
*Neopreptetos arusettae*	*Pomacanthus sexstriatus*		FJ788490	(53)
*Bianium arabicum*	*Lagocephalus lunaris*	MH157054	MH157076	(31)
*Bianium plicitum*	*Torquigener squamicauda*, *Sphoeroides testudineus*	MH157056	MZ345682	(31, 49)
*Diploproctodaeum cf. monstrosum*	*Arothron hispidus*	MH157059	FJ788473	(31, 53)
*Diploproctodaeum momoaafata*	*Lagocephalus lunaris*	PP941784	FJ788474	(59)
*Neohypocreadium dorsoporum*	*Chaetodon flavirostris*	AF392443	FJ788487	(55, 60)
*Deraiotrema platacis*	*Platax pinnatus*	MN073840	MN073841	(58)
*Diplocreadium tsontso*	*Balistoides conspicillum*	FJ788472	FJ788472	(53)
*Echeneidocoelium indicum*	*Echeneis naucrates*	FJ788475	FJ788475	(53)
*Hypocreadium lamelliforme*	*Balistes capriscus*		MZ345680	(49)
*Hypocreadium myohelicatum*	*Balistes polylepis*		MK648295	(61)
*Hypocreadium patellare*	*Balistoides viridescens*		FJ788478	(53)
*Hypocreadium picasso*	*Rhinecanthus aculeatus*		FJ788479	(53)
*Hypocreadium toombo*	*Pseudobalistes fuscus*		FJ788480	(53)
*Lepidapedoides angustus*	*Epinephelus cyanopodus*		FJ788482	(53)
Outgroups				
*Austroholorchis sprenti*	*Sillago ciliata*	MH157065	MH157075	(31)
*Aephnidiogenes major*	*Diagramma labiosum*		FJ788468	(53)
*Holorchis castex*	*Diagramma picta*		FJ788476	(53)
*Holorchis gigas*	*Plectorhinchus chrysotaenia*		FJ788477	(53)

In bold new sequences obtained in the present study.

Three phylogenetic trees were constructed: two using ITS2 and partial 28S rDNA regions for Lepocreadiidae, and one using partial 18S rDNA for Hemiuridae (see species and outgroups data in Tables 1, 2). Alignments of individual regions were performed using MAFFT (available at http://www.ebi.ac.uk/Tools/msa/mafft/) and MEGA X (16). Phylogenetic and molecular evolutionary analyses were conducted on the aligned nucleotide sequences and inferred by both the maximum-likelihood (ML) method using W-IQTREE online software (17) and by Bayesian inference (BI) using BEAST v1.8.0 (18). To determine the evolutionary model that best fits our dataset, jModeltest 2.1.1 (19) was used, with model selection based on AIC. The results indicated that the general time reversible model with an estimate of gamma distribution among-site rate variation and invariant sites (GTR + G + I) was the most appropriate. The number of substitution types was fixed at 6. The 4 × 4 substitution model was used, while rate variation across sites was modeled with a gamma distribution. Node supports were evaluated using the ultrafast bootstrap with 1,000 replicates (20). The percentage of trees in which the associated taxa clustered together is shown next to the branches. Four Markov Chain Monte Carlo (MCMC) chains were run for 10,000 generations, with sampling performed every 10 generations, with the first 250 sampled trees discarded as burn-in. Finally, a 50% majority rule consensus tree was constructed. The ITS2 tree for Lepocreadiidae involved 56 sequences with a total of 506 positions in the final dataset. The 28S tree for Lepocreadiidae involved 75 sequences with a total of 1,369 positions in the final dataset. The 28S tree for Hemiuridae involved 42 sequences with a total of 1,339 positions in the final dataset. For the ITS2 sequences of Lepocreadiidae, evolutionary divergence was calculated using p-distance with 1,000 bootstrap replicates in MEGA X.

## 3 Results

Three digenean genera of trematodes were morphologically identified: *Lecithocladium* Lühe, 1901 (Hemiuridae) (21) from the short mackerel *R. brachysoma* (Scombridae) (prevalence 77%, *N* = 22); *Prodistomum* Linton, 1910 (22) from *R. brachysoma* (prevalence 18%, *N* = 22) and the Indian mackerel *Rastrelliger kanagurta* (Cuvier, 1816) (prevalence 100%, *N* = 1) (Scombridae); and *Lepocreadioides* Yamaguti, 1936 (Lepocreadiidae) (22) from the double-lined tonguesole *Paraplagusia bilineata* (Bloch, 1787) (Cynoglossidae) (prevalence 75%, *N* = 4).

**Figure 1:**
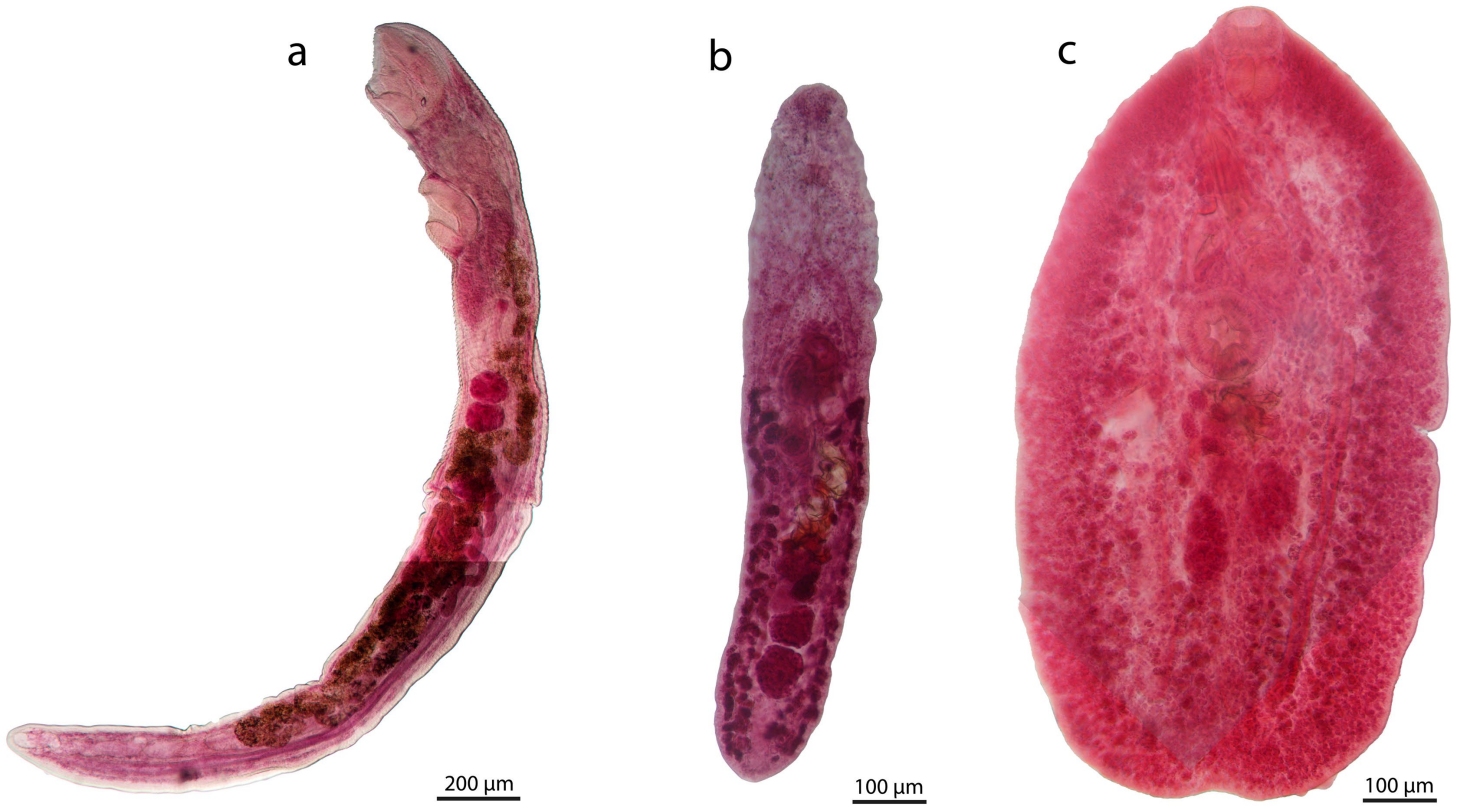
Microphotographs of the three studied trematodes. **(a)** *Lecithocladium angustiovum***, (b)** *Prodistomum* sp. and **(c)** *Lepocreadioides orientalis*.

**Figure 2:**
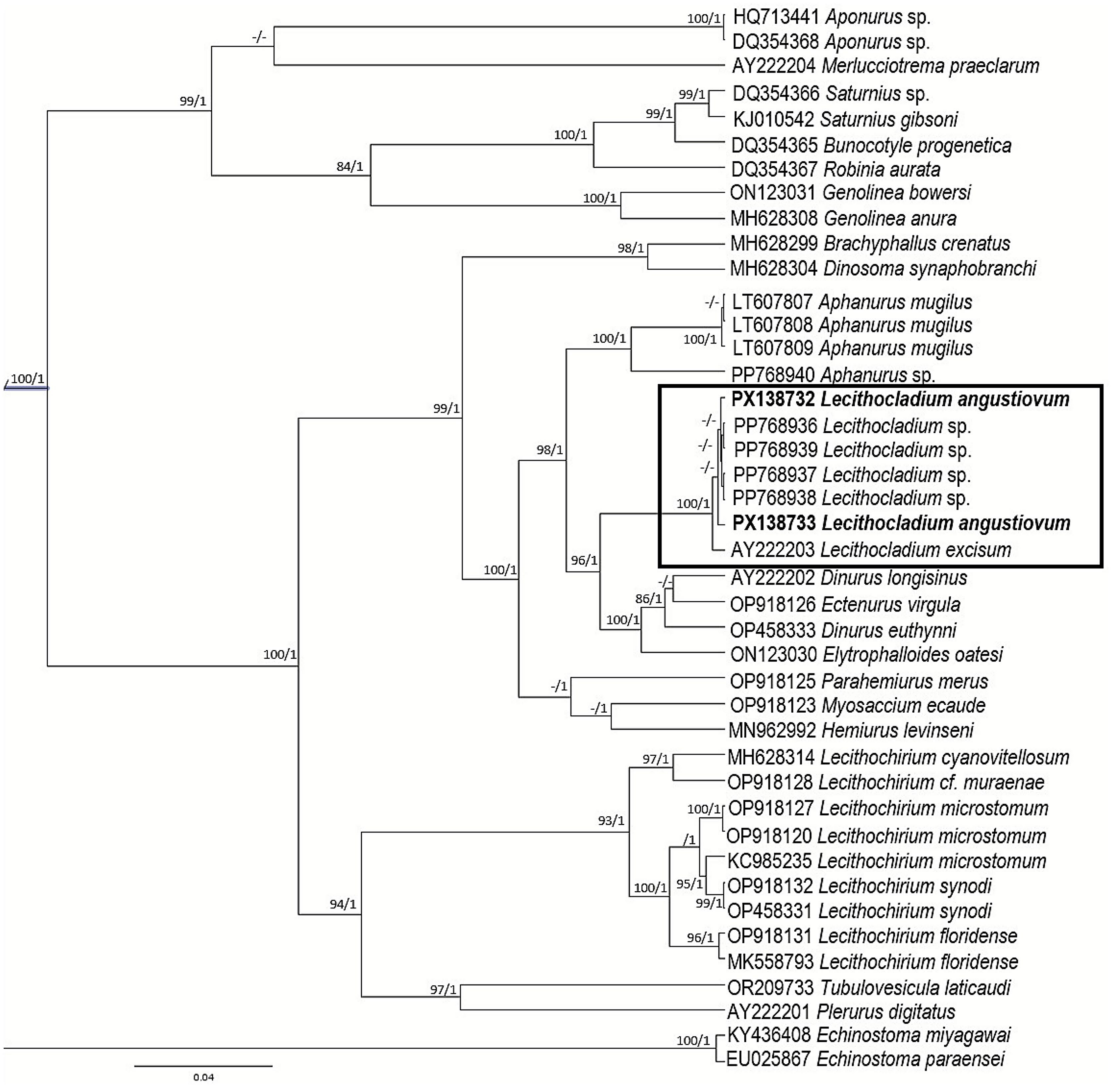
Phylogenetic tree for Hemiuridae species based on Bayesian inference and maximum likelihood from the 28S dataset. Strongly supported nodes (Bayesian posterior probabilities >0.8 and maximum likelihood bootstrap values >80) are indicated by filled circles. The scale bar indicates the expected number of substitutions per site.

The hemiurid recorded in this study agrees in morphology and morphometry with *Lecithocladium angustiovum* Yamaguti, 1953 (12). It presents an elongated body, long ecsoma (approximately the same size as the body length), cuticular denticulations over the entire body surface, a terminal, cup-shaped oral sucker, and a uterus extending into the ecsoma for more than half of its length (Figure 1a). Six sequences of *Lecithocladium* were obtained: PX138719-20 (18S), PX138820-21 (ITS2), and PX138732-33 (28S). The 18S sequences of the species are identical and match 100% with *L. angustiovum* sequences available in GenBank (KY498584-85). The 28S tree indicates that this species is the same as that previously recorded in *R. brachysoma* from the Gulf of Thailand (5), and these sequences form a well-supported clade (96/1) with *Lecithocladium excisum* (Rudolphi, 1819) Lühe, 1901 (Figure 2), forming a monophyletic group.

Morphological characteristics of the lepocreadiid specimens found in *R. brachysoma* and *R. kanagurta* are consistent with the diagnosis of *Prodistomum* Linton, 1910 (10) (Figure 1b). These specimens present an elongate body, slightly wider anteriorly, measuring 816.29 (573.73–1006.74) in length and 155.83 (86.77–202.39) in width. The tegument is completely spinous, and eye-spot pigment is scattered in the anterior part of the forebody. The oral sucker is terminal and cup-shaped, measuring 48.57 (36.77–57.58) in length and 47.32 (34.92–72.86) in width. The ventral sucker is rounded to oval, measuring 70.33 (63.02–85.26) in length and 66.51 (53.71–80.83) in width. The sucker-width ratio is 1:1.3 (1.1–1.5). The forebody is 336.65 (216.38–418.52) long [41 (37–43)% of body length]. The prepharynx is 51.33 (35.00–67.74) long, and the pharynx is oval, measuring 32.16 (21.13–39.74) in length and 26.03 (17.05–37.04) in width. The oesophagus and pseudoesophagus are not differentiable, measuring 34.76 (18.00–52.28) in length. The intestinal bifurcation is located in the posterior third of the forebody, and the caeca terminate is close to the posterior end. The two testes are ovals and arranged in tandem; the anterior testis is 69.84 (55.19–83.30) long and 54.58 (40.17–66.86) wide, and the posterior testis is 74.86 (65.31–90.63) long and 57.74 (46.21–85.92) wide. The cirrus-sac is elongate, measuring 144.57 (91.16–189.87) long and 54.53 (48.17–61.88) wide, and it is located in the first part of the hindbody. The internal seminal vesicle is oval, and the external seminal vesicle is coiled and measures 74.34 (34.58–98.02) in length. The genital atrium is large and diverticulate, and the genital pore is close to the antero-sinistral edge of the ventral sucker. The ovary is oval and pre-testicular, measuring 55.59 (44.54–73.48) in length and 43.26 (32.97–56.71) in width. The seminal receptacle is saccular and is located between the ovary and the anterior testis. The uterus is pre-ovarian, intercaecal, and mainly posterior to the external seminal vesicle. Eggs are tanned and operculate, measuring 49.45 (41.18–57.05) in length and 31.11 (24.22–47.09) in width. The vitellarium is follicular and extends close to the posterior margin of the ventral sucker, and the excretory pore is terminal.

**Figure 3:**
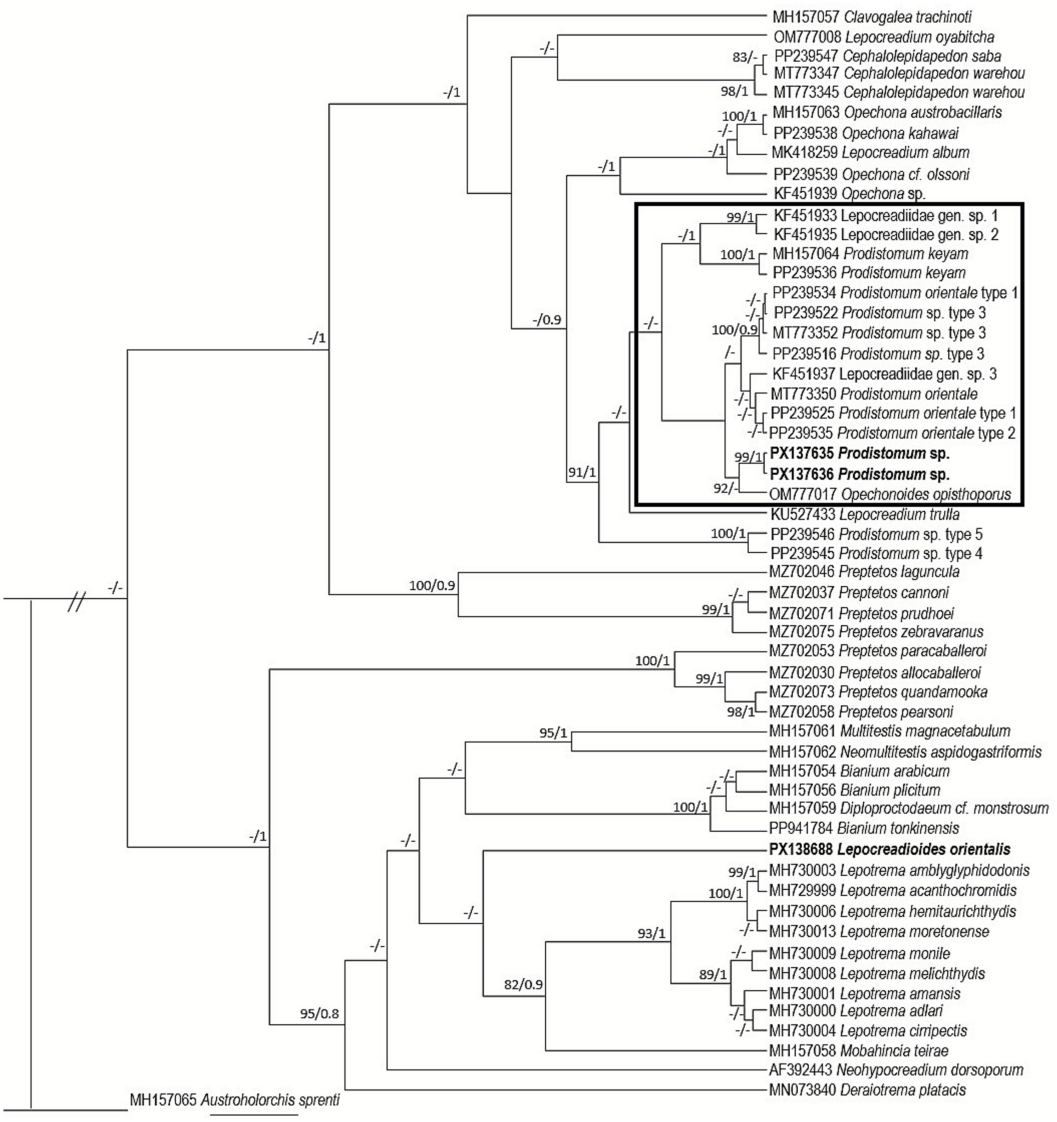
Phylogenetic tree for Lepocreadiidae species based on Bayesian inference and maximum likelihood from the ITS2 dataset. Strongly supported nodes (Bayesian posterior probabilities >0.8 and maximum likelihood bootstrap values >80) are indicated by filled circles. The scale bar indicates the expected number of substitutions per site.

**Figure 4:**
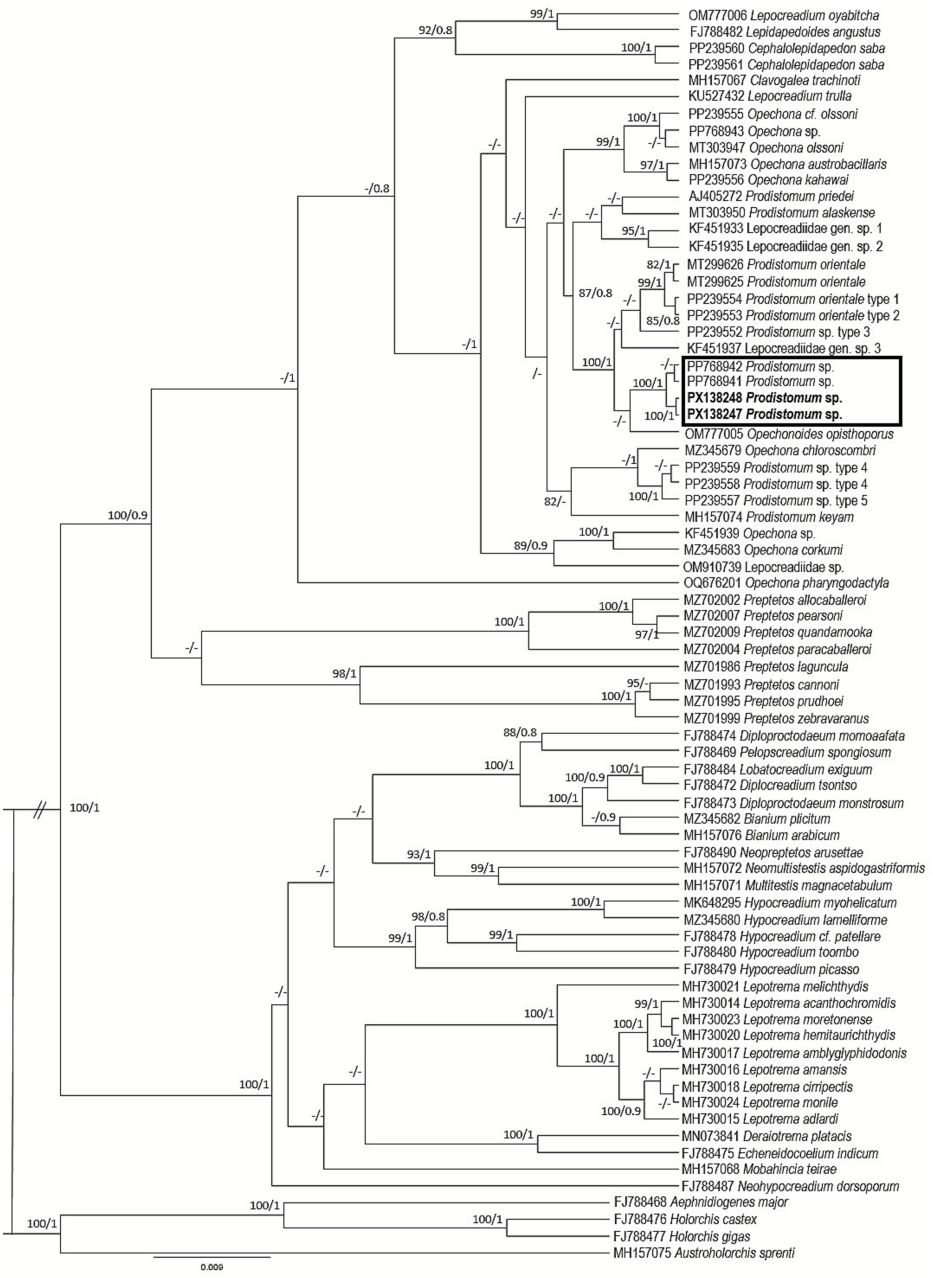
Phylogenetic tree of Lepocreadiidae species based on Bayesian inference and maximum likelihood from the 28S dataset. Strongly supported nodes (Bayesian posterior probabilities >0.8 and maximum likelihood bootstrap values >80) are indicated by filled circles. The scale bar indicates the expected number of substitutions per site.

Six sequences of *Prodistomum* were obtained: PX136578-79 (18S), PX137635-36 (ITS2), and PX138247-48 (28S). The ITS2 and 28S trees showed a well-supported clade (100/1) for *Prodistomum* spp., *Opechonoides opisthoporus* Duong et al. (23), and other Lepocreadiidae spp. (Figures 3, 4). The species recorded in the study is close to *O. opisthoporus* (genetic distance of ITS2 is 1.7%). The genetic distance from other *Prodistomum* spp. varies between 0.9 and 3.9%, with the closest similarity to *Prodistomum orientale* (Layman, 1930) (24) type 1 and type 2 (0.9%). Cribb et al. (13) differentiated *Prodistomum* spp. infecting fish from Japan and Australia according to pharynx size and vitellarium extension. The species described in this study is more similar to *P. orientale* type 1 in having a smaller pharynx (<44 μm long), but more similar to *P. orientale* type 2 in the extension of the vitelline follicles, which reach much closer to the ventral sucker. Additionally, *Prodistomum* sp. described here is significantly smaller (body length 800 μm) than *P. orientale* type 1 (1,600 μm) and *P. orientale* type 2 (1,700 μm). The 28S tree indicates that the species studied here is the same as that recorded in *R. brachysoma* (1 bp different) from the Gulf of Thailand (5).

Our individuals of *Lepocreadioides* were classified as *Lepocreadioides orientalis* Park, 1939, following the descriptions of Bray and Cribb (10). These specimens are characterized by a broadly oval body with somewhat wrinkled margins; oral sucker and pharynx subglobular; genital pore sinistral at the level of the middle of the oral sucker; ovary tri-lobed and pre-testicular; seminal receptacle large, oval, and medio-dorsal to the ovary (Figure 1c). Two sequences of this species were obtained: PX138257 (18S) and PX138688 (ITS2). Until recently, there have been no available sequences of this genus. The ITS2 tree does not show a well-supported relationship for our sequence; it is placed close to *Lepotrema* spp. but with high genetic distance (7–8%) (Figure 3).

## 4 Discussion

The hemiurid *L. angustiovum* was originally described from *R. kanagurta* in the waters of Sulawesi (formerly Celebes) (25). It has subsequently been reported in mackerels of the genus *Rastrelliger* (Scombridae) (26, 27). More recently, this genus of digenean was recorded in the short mackerel (*R. brachysoma*) from the Gulf of Thailand, reported as *Lecithocladium* sp. (5), and later characterized to species level as *L. angustiovum* using 18S rDNA in Indonesia (12). The short mackerel, which accounts for approximately half of the total value of Thailand’s marine fisheries and is the country’s most economically important fish species, has experienced a dramatic decline in catches since 2013 due to overfishing (28). Consequently, this species has attracted considerable research interest, including helminthological studies. Our phylogenetic analyses support the presence of *L. angustiovum* in the short mackerel in the Gulf of Thailand, corresponding to the same species previously recorded (5). Even if this species had been recorded earlier in this area, our study contributes new molecular data.

*Prodistomum orientale* has been reported in India from the same host species examined in the present study (*R. brachysoma* and *R. kanagurta*), although without accompanying molecular data (27). This digenean shows a preference for scombrid hosts (24) and has been reported, but not described, in the Gulf of Thailand from *R. brachysoma* (5) as *Prodistomum* sp., clustering with *P. orientale*. Our molecular phylogeny based on 28S rDNA indicates that the species studied here is the same taxon reported by (5), differing by only 1 bp. Cribb et al. (13) studied several *Prodistomum* spp., including *P. orientale* type 1 and type 2 in Australia and Japan, respectively, and *Prodistomum* sp. type 3 in Australia. The ITS2 sequences of *P. orientale* type 1 and type 2 were identical to *P. orientale* from *Scomber australasicus* Cuvier, 1832 (29) and differed from *Prodistomum* sp. type 3 by 1–2 bp. The ITS2 sequence of the species studied here differs by 5 bp from *P. orientale* and by 7 bp from *Prodistomum* sp. type 3, clearly indicating that it belongs to a different species. However, this species presents several morphological differences, including a uterus that overlaps with the testes, short caeca, a long excretory vesicle containing corpuscles, and oblique testes (23). In summary, this study contributes both a morphological description and new molecular data.

The digenean *Lepocreadioides orientalis* Park, 1939 was originally described in the Yellow Sea of North Korea as *Cynoglossus joyneri* Günther, 1878 (Cynoglossidae) (30). This species was later recorded from India in the Arabian Sea (Kerala coast) in four species of *Cynoglossus* (30). In Australia (10), it was reported in *Cynoglossus bilineatus* (Lacepède, 1802). According to these authors, this digenean has been reported in Cynoglossidae (15 species), Soleidae (3 species), Platycephalidae (2 species), and Sciaenidae (1 species). The study by Bray et al. (31) stated the following: “This species has not been re-collected from Moreton Bay since the report from the four-lined tonguesole *C. bilineatus* (Lacepède, 1802) by Bray and Cribb (10), and no material is available for molecular characterization.” The present study provides the first molecular sequences and phylogeny for this parasite; in addition, we report the first record from the Gulf of Thailand. However, the phylogenetic results are not very conclusive. In this study, only 18S and ITS2 sequences were obtained. Using BLAST, our 18S sequences showed high similarity (98%) with species from different genera (*Preptetos caballeroi* Pritchard, 1960; *Lepocreadium trulla* (Linton, 1907) Linton, 1910; *Opechona* sp.) or with species lacking taxonomic identification at the genus level. According to the ITS2 tree, this species is closest to the monophyletic clade of *Lepotrema* spp., but with a high genetic distance.

Another aspect to consider is the taxonomy of the hosts in which these trematodes are reported, as there have been revisions in fish species classification, their phylogenetic relationships, nomenclature, and further upcoming rearrangements in host taxonomy [e.g., (32), in the case of the genus *Rastrelliger*. A better understanding of host phylogenetic relationships would help clarify parasite specificity.

Finally, our findings may contribute to understanding fish migratory patterns (28), biogeographic barriers (33), and environmental conditions in the Gulf of Thailand, particularly if future studies examine the same host species in other geographical regions. In conclusion, this study provides new data on the helminths of marine fishes from the Gulf of Thailand, but further research is needed to provide a more comprehensive understanding.

## Data Availability

The datasets presented in this study can be found in online repositories. The names of the repository/repositories and accession number(s) can be found in the article/supplementary material.
